# CCBuilder 2.0: Powerful and accessible coiled‐coil modeling

**DOI:** 10.1002/pro.3279

**Published:** 2017-09-15

**Authors:** Christopher W. Wood, Derek N. Woolfson

**Affiliations:** ^1^ School of Chemistry University of Bristol, Cantock's Close Bristol BS8 1TS United Kingdom; ^2^ School of Biochemistry University of Bristol, Medical Sciences Building, University Walk Bristol BS8 1TD United Kingdom; ^3^ BrisSynBio, University of Bristol, Life Sciences Building, Tyndall Avenue Bristol BS8 1TQ United Kingdom

**Keywords:** coiled coil, collagen, computational design, structural modeling, structural bioinformatics, web app, parametric design, protein design, synthetic biology

## Abstract

The increased availability of user‐friendly and accessible computational tools for biomolecular modeling would expand the reach and application of biomolecular engineering and design. For protein modeling, one key challenge is to reduce the complexities of 3D protein folds to sets of parametric equations that nonetheless capture the salient features of these structures accurately. At present, this is possible for a subset of proteins, namely, repeat proteins. The α‐helical coiled coil provides one such example, which represents ≈ 3–5% of all known protein‐encoding regions of DNA. Coiled coils are bundles of α helices that can be described by a small set of structural parameters. Here we describe how this parametric description can be implemented in an easy‐to‐use web application, called CCBuilder 2.0, for modeling and optimizing both α‐helical coiled coils and polyproline‐based collagen triple helices. This has many applications from providing models to aid molecular replacement for X‐ray crystallography, *in silico* model building and engineering of natural and designed protein assemblies, and through to the creation of completely *de novo* “dark matter” protein structures. CCBuilder 2.0 is available as a web‐based application, the code for which is open‐source and can be downloaded freely. http://coiledcoils.chm.bris.ac.uk/ccbuilder2.

**Lay Summary:**

We have created CCBuilder 2.0, an easy to use web‐based application that can model structures for a whole class of proteins, the α‐helical coiled coil, which is estimated to account for 3–5% of all proteins in nature. CCBuilder 2.0 will be of use to a large number of protein scientists engaged in fundamental studies, such as protein structure determination, through to more‐applied research including designing and engineering novel proteins that have potential applications in biotechnology.

**Abbreviations**
BUDE: Bristol University Docking Engine
BUFF: Bristol University docking engine Force Field
ISAMBARD: Intelligent System for Analysis, Model Building And Rational Design

## Introduction

Protein design has advanced rapidly over the past decade, with biomolecular modeling making a significant contribution to this development.^1^, ^2^, ^3^, ^4^, ^5^, ^6^ An exciting and emerging aspect of protein design centers on exploring the “dark matter” of protein fold space; that is, those protein folds that are theoretically possible but have not been observed in or explored by nature.^7^ This offers near unlimited potential for designing new protein structures that could have a range of applications in synthetic biology and biotechnology.^7^, ^8^ However, designing such *truly de novo* structures represents a significant challenge, as no specific sequence or structural data are available as guides. An additional and general challenge in protein design is the combinatorial nature of the problem, which renders unguided searches through sequence and structural space impossible. Computational methods are required to address both of these challenges. Parametric modeling and design of protein structures^9^, ^10^, ^11^, ^12^—i.e., where a fold or set of related folds are described geometrically—offers a route to addressing both these challenges as it effectively guides and reduces the conformational space examined.

In order for parametric modeling to be useful and effective, the fold must have a regular structure that can be described using a small number of mathematical parameters. Repeat proteins are an excellent target for this type of modeling.^13^ The α‐helical coiled coil is one such example, which has been fertile ground for initial exploration into parametric modeling and design.^4^, ^9^, ^10^, ^14^, ^15^

**Figure 1:**
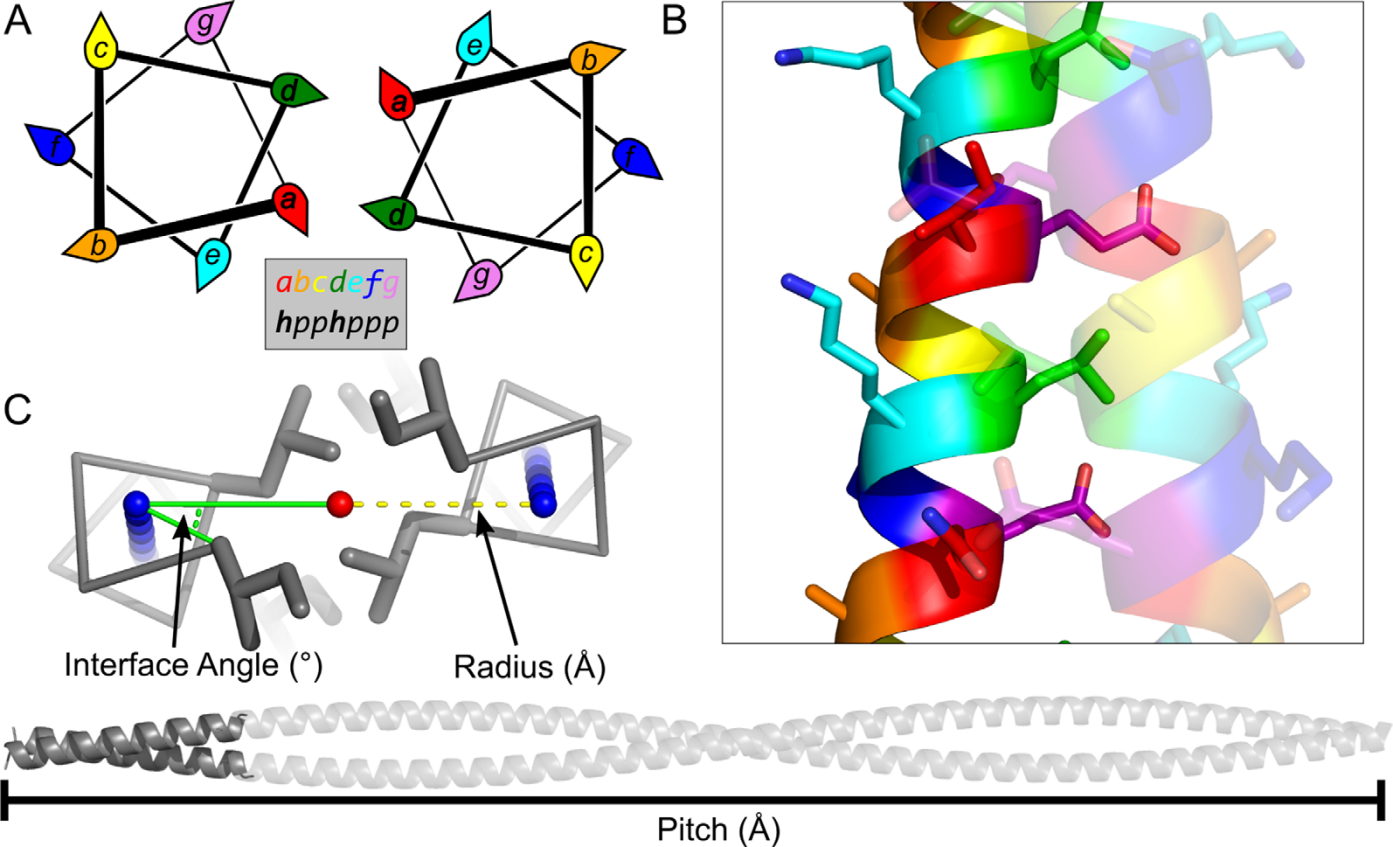
Structure of α‐helical coiled coils. (A) Helical‐wheel diagrams showing the projection of residues in the heptad repeat. (B) Helices in a coiled coil pack closely together, forming knobs‐into‐holes interactions. (C) Coiled coils can be described using three geometric parameters: interface angle (°), radius (Å), and pitch (Å).

Coiled coils account for between 3% and 5% of all protein‐encoding DNA.^16^, ^17^ They perform a diverse range of natural functions including structural roles, DNA binding, and driving protein–protein interactions.^18^, ^19^, ^20^ This ubiquitous fold consists of two or more α helices that wrap around each other to form a rope‐like, superhelical structure.^21^, ^22^ Classical coiled‐coil helices are amphipathic with a hydrophobic stripe along their length. This is usually generated through a repeating sequence unit of seven amino acids known as a heptad repeat, although other repeats are possible and observed.^22^, ^23^ Conventionally, the residues of the heptad are assigned the letters *a*–*g* (Fig. 1). Hydrophobic residues usually occupy the first (*a*) and fourth (*d*) sites of the repeat, and the number, precise position, and type of the hydrophobic side chain strongly influences the oligomeric state, topology, and partnering preference of the coiled coil.^10^, ^20^, ^21^, ^22^, ^24^, ^25^, ^26^

Most of the binding enthalpy that drives assembly of the helices comes from the formation of a hydrophobic interface, with lesser contributions from salt‐bridge and/or hydrogen‐bonding interactions (Fig. 1).^27^ The formation of the hydrophobic interface is facilitated by an intimate mode of helix‐helix interaction known as knobs‐into‐holes packing. This is formed when a series of “knob” residues on one helix projects into complementary “holes” formed by 4‐residue diamonds on a partnering helix.^21^

Dimeric, trimeric, and tetrameric coiled coils are the most abundant forms of α‐helical coiled coils found in nature, accounting for >98% of all known coiled coils.^28^, ^29^ This wealth of information has led to reliable methods for predicting these low‐order, or classical coiled‐coil states from sequence.^22^, ^23^, ^30^, ^31^, ^32^ Moreover, these structures and associated sequences provide rules of thumb for the successful rational *in biro* design of simple coiled‐coil assemblies.^33^, ^34^ In addition, there are examples of both natural and designed coiled coils with higher oligomeric states.^9^, ^10^, ^35^, ^36^, ^37^ α‐Helical barrels—i.e., coiled coils with oligomeric states >4—are of particular interest as they have channels that run along their lengths. These structures have a range of potential applications as scaffolds for *de novo* enzymes, materials, and membrane channels.^3^, ^38^, ^39^, ^40^ As higher order coiled coils are so rare in nature, atomistic modeling is essential for the design of these structures. This presents a considerable opportunity for *in silico* modeling and design to cover and test a larger sequence space ahead of time‐consuming experiments.

## Parametric Modeling of Coiled‐Coils

Owing to the regular nature of α‐helical coiled coils, their structures can be described using a small number of geometric parameters. The first mathematical parameterization was provided by Crick in 1953, who described the super helix of a coiled coil using three simple parameters pitch (*P*) or pitch angle (*α*), radius of the assembly (*r*), and the interface angle (also known as the Crick angle at *a* or *φCα*) (Fig. 1).^41^

A range of software tools have applied or built upon Crick's original parameterization: In 1995, Offer and Session developed software (MakeCCSC) to model the backbone of the coiled coil, utilizing Crick's original parameterization.^42^ Around the same time, Harbury *et al*. also implemented Crick's equations to aid the design of a right‐handed coiled‐coil tetramer.^43^, ^44^ A generalization of the Crick equations followed in 2002, allowing coiled coils with noncanonical repeats to be modeled.^45^ More recently, Grigoryan and DeGrado have developed methods for fitting Crick parameters to structural data of coiled coils, and building backbone models.^15^

We have added to this body of work by creating CCBuilder,^14^ a web‐based application for generating models of coiled coils and by developing the ISAMBARD software package,^46^ which generalizes parametric model building, and has a range of tools for modeling α‐helical coiled coils, helical bundles and, indeed, any parameterizable protein fold.

## CCBuilder

CCBuilder was developed with an emphasis on usability and to be accessible to nonspecialist users. Given a sequence and a few structural parameters, it will create a fully atomistic model of a coiled coil. The pipeline for generating a model is straightforward: a modified version of MAKECCSC generates a backbone model with the required structural parameters; the model is passed to Rosetta (using the PyRosetta interface) to pack the side chains^47^; CCBuilder then runs a range of analysis programs to assess the quality of the model, including geometric evaluation of the backbone, knobs‐into‐holes analysis with SOCKET,^48^ and interaction energies using two all‐atom force fields, BUDE^49^, ^50^ and Rosetta.^51^

Previously, we tested the accuracy of CCBuilder's modeling protocol by recreating the structures of 653 coiled‐coil proteins with known structures.^14^ These had a range of common and unusual topologies, which accounted for >97% of all experimental determined coiled‐coil structures. The models were shown to be highly accurate, with respect to the experimental structures [0.77 Å (standard deviation 0.49 Å) backbone RMSD], demonstrating that a parameterization with only 3 or 4 structural parameters can capture much of the natural complexity of this class of protein fold, including rotameric preference of side chains.^14^

Our focus on usability of CCBuilder has enabled wide spread adoption by many users, not just by specialists in molecular modeling or protein design, and for many applications. In a similar vein to Crick's original motivation to parameterize the α‐helical coiled coil, we routinely use models generated by CCBuilder to phase X‐Ray crystal structure data during molecular replacement.^10^ Similarly, others have used models generated with CCBuilder to fit SAXs data^52^, ^53^ or model electron microscopy data.^38^, ^53^, ^54^ Various natural coiled‐coil sequences, with unknown structures, have been analyzed using models produced by CCBuilder: models have been used to predict of the sequence register of the heptad repeat^55^; to probe the interaction energy of putative interfaces^56^, ^57^; and to calculate electrostatic surface potentials.^58^ Furthermore, models from CCBuilder have been used as the basis for other computational methods, such as homology modeling, using other software packages.^59^, ^60^

## CCScanner

The CCBuilder protocol was further developed to create CCScanner, which automates model building by fitting coiled coil parameters for a given sequence. To do this, the model‐building protocol was extracted from CCBuilder, and a genetic algorithm was implemented to optimize the structural parameters used to build the model. While CCScanner is unpublished, we have incorporated its model‐building methodology into the ISAMBARD software package.^46^

CCScanner can be used to predict the oligomeric state and coiled‐coil parameters for a given sequence, whether natural or designed. We applied it as part of the computational design of α‐helical barrels.^10^ High‐resolution X‐ray crystal structures were determined for four peptides—CC‐Pent, CC‐Hex2, CC‐Hex3, and CC‐Hept—using models produced by CCScanner to phase the data during molecular replacement. CCScanner accurately predicted the oligomeric state and coiled‐coil parameters for all of the structurally characterized α‐helical barrels, demonstrating that it is a powerful tool for the design of coiled coils in both observed and possible conformations. These peptides extend a basis set of completely *de novo* coiled coils,^27^ which now contains a de novo designed dimer through to heptamer (Fig. 4).

## CCBuilder 2.0

Since the original development of CCBuilder, the architecture of the application has proven difficult to maintain, so it has become increasingly necessary to update the application. Furthermore, with developments in coiled‐coil design, there is demand to generate models on a much higher scale than previously possible, using optimization algorithms similar to those developed for CCScanner. Here we present CCBuilder 2.0, a complete rewrite of the CCBuilder web application using modern web technologies and software design tools/principles, and state‐of‐the‐art parametric modeling software. This brings a range of new features and improvements to robustness, usability, scalability, and portability. The CCBuilder 2.0 web application can be accessed at: http://coiledcoils.chm.bris.ac.uk/ccbuilder2. The source code is open source and available to download from the Woolfson Group GitHub repository (https://github.com/woolfson-group).

### Architecture

**Figure 2:**
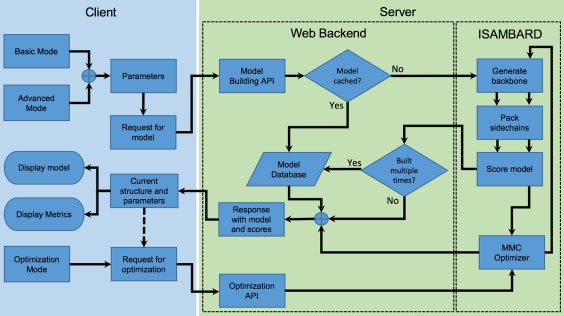
Architecture of the CCBuilder 2.0 web application. The client side (Elm, JavaScript, HTML, and CSS) is used for submitting parameters and displaying models/metrics. The web backend (Flask, MongoDB, uWSGI, and NGINX) serves the application web pages and also provides an RESTful API to the modeling engine (implemented using ISAMBARD).^46^

CCBuilder 2.0 has three main software layers: the user interface; an RESTful API backend; and the model building protocols. Each layer has been designed to be as independent as possible (Fig. 2), to make the platform flexible.

The user interface to the web application is written in Elm, JavaScript, HTML, and CSS. Elm is a functional programming language specifically for building web applications. It compiles down to native JavaScript and offers excellent performance without runtime exceptions (http://elm-lang.org/).

**Figure 3:**
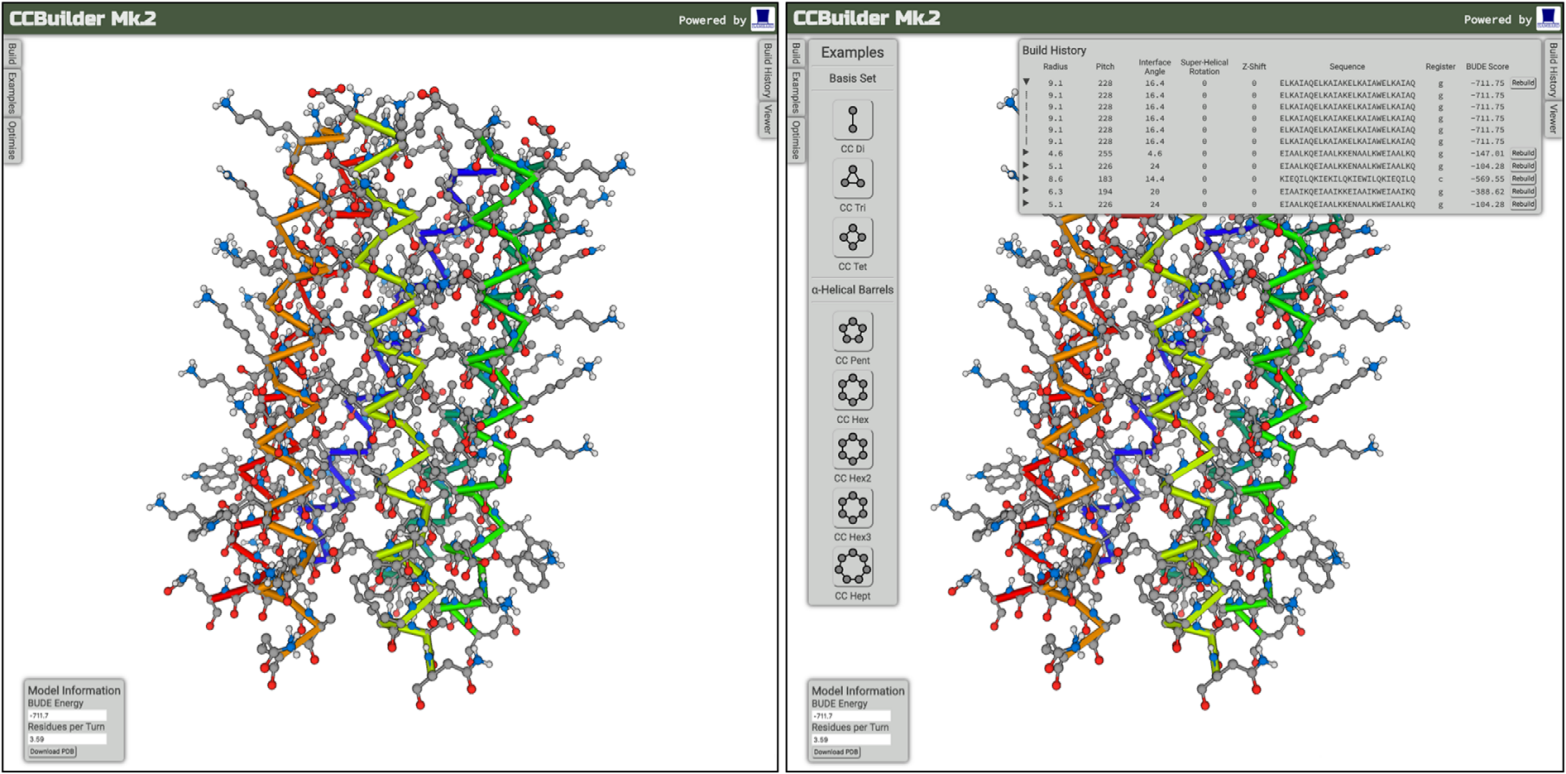
The CCBuilder 2.0 Interface. Panels can be hidden to give a full view of the model in the molecular viewer.

The main user interface provides a molecular viewer for visualizing models and panels for inputting parameters, building example models, running parameter optimizations, displaying model information, downloading the structure, showing build history, and controlling the viewer (Fig. 3).

**Figure 4:**
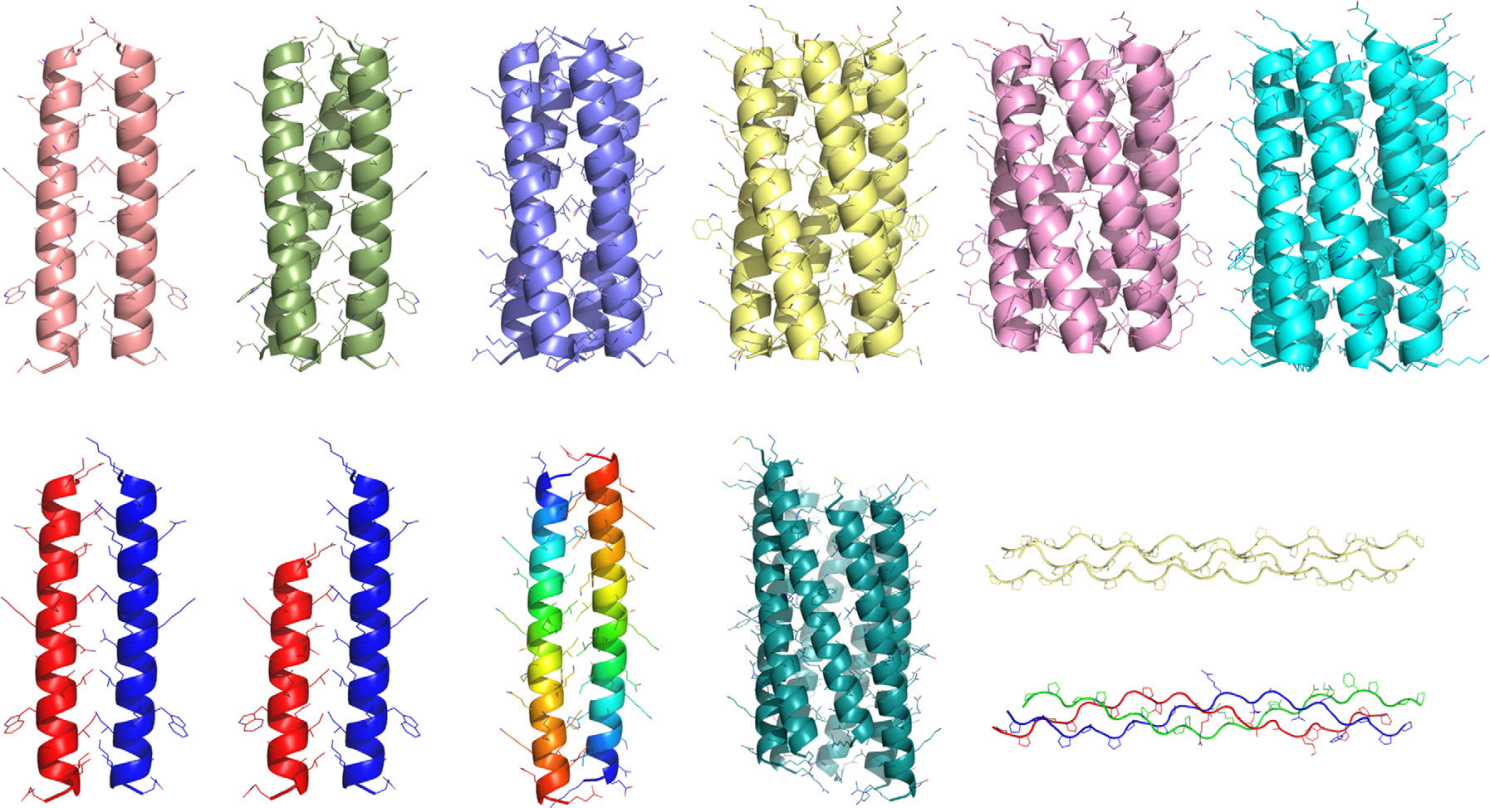
CCBuilder 2.0 can model a diverse range of coiled coils and collagens. Top row: dimer, trimer, tetramer, pentamer, hexamer, and heptamer, which are all homotypic. Bottom row: A4/B4 heterodimer, A3/B4 heterodimer, antiparallel homodimer, slipped heptamer, and homotrimeric and heterotrimeric collagens.

The front‐end collects and validates user input before generating and sending an HTTP request to the backend API (Fig. 2). With this architecture, any service could supply the model as long as the HTTP response is formatted correctly, allowing the backend model building protocols to remain up‐to‐date with the main ISAMBARD repository. The RESTful API is designed to allow researchers to generate models with the CCBuilder 2.0 server programmatically, increasing the number of models that they can generate.

The backend of the website is written in Python using the Flask web framework (http://flask.pocoo.org/). It is very lightweight, providing basic routing, templating for a few web pages and, most importantly, providing an RESTful API for building, saving, and retrieving models. The application is served using uWSGI with NGINX.

The model building protocol takes the parameters provided by the front end, as JSON in the HTTP request body, and passes them to a model building script (Supporting Information). The ISAMBARD software package is used to build the model.^46^ This means that if user requires more complex or a larger number of models they can bypass the web app and use ISAMBARD to run this model building protocol on their local machine.

Once a model has been generated the structure and scoring metrics (BUDE interaction energy, knobs‐into‐holes analysis, and backbone properties) are returned as JSON in an HTTP response, which is decoded and displayed by the front end. The structure is shown using a molecular viewer embedded in the web app, which is built using PV, a WebGL‐based visualizer for proteins and biomolecules.^61^

When a model is built, the backend stores the parameters used to create a model in a database (MongoDB), and if a model is requested repeatedly, then the model and scoring data are cached and then subsequently served directly. This reduces server load and improves the overall user experience.

To provide portability and scalability, the whole application has been created to run in three Docker containers, one container for the web and modeling elements (NGINX, uWSGI, Flask, and ISAMBARD) and one for managing optimization jobs and another for the database (MongoDB). A Docker Compose script is included in the application repository, allowing users to easily run the web application locally.

### The interface

The CCBuilder 2.0 interface brings to focus the most important element of the application: the model itself. The window is scalable and almost all of it is dedicated to displaying the model, with floating panels that can be hidden. There are six such panels, which are used for submitting parameters, selecting examples, optimizing models, displaying model information, displaying build history, and tweaking the representation of the model in the molecular viewer. The state of the app is cached in local storage every time a model is built, allowing users to resume from exact point where they left off in the previous sessions.

### Building a model

The model building protocol in CCBuilder 2.0 is implemented entirely using the ISAMBARD software package.^46^ It employs a generalized parametric description of a coiled coil, which is much more flexible and offers increased model building accuracy over the methodology employed by the original CCBuilder (*vida supra*). Side chains are packed onto the backbone structure using an interface that ISAMBARD provides to SCWRL4.^62^ There are a range of analysis modules included within ISAMBARD including tools for performing geometric analysis of the backbone and a modern implementation of the SOCKET protocol.^48^ The BUFF module in ISAMBARD provides an all‐atom scoring potential, and is a standalone implementation of the BUDE force field.^49^, ^50^

To create a model using CCBuilder 2.0, users can get started quickly with a range of example models that recreate known coiled‐coil structures. These can be used as a starting point for modeling coiled coils with an unknown structure. From there, the user provides parametric values that describe the coiled‐coil architecture. In the basic building mode, the user gives a single value for the oligomeric state, radius, pitch, interface angle, and sequence (with an associated register). As a consequence, this mode can only be used to model parallel, homo‐oligomeric coiled coils. The advanced mode allows the user to break symmetry by specifying values for all of these parameters independently for each helix. Furthermore, it allows the user to specify some additional parameters such as helix orientation, z‐shift, and super‐helical rotation, allowing antiparallel and slipped systems to be modelled. The super‐helical rotation value refers to deviation from the ideal value for a symmetric bundle, which are calculated with the following equation:

$$i-1\left(\frac{360}{n}\right)$$

where *i* is the helix identifier—e.g., helices 1, 2, 3, and 4 in a tetrameric coiled coil—and *n* is the oligomeric state. For example, in a tetramer, helix 1 would have a default super‐helical rotation value of 0°, while helix 3 would have a value of 180°.

All parameters are defined relative to the super‐helical axis of the coiled coil, and so are completely decoupled from one another. This is required for users to generate models of complex coiled coils, where symmetry is broken and the concept of a reference helix becomes meaningless.

CCBuilder 2.0 offers a significant improvement in parameter submission and validation. CCBuilder was restricted to a maximum of 8 helices in the advanced build mode. With the design and discovery of larger coiled‐coil assemblies, this restriction has been lifted. Tools have been added to facilitate building models of coiled coils with higher oligomer states, such as “copy and paste” buttons to allow parameters to be transferred between helices, meaning that repetitive parameter entry is avoided. Users can switch between the basic and advanced building modes at any time, and parameters that have been entered up to that point are carried over. For example, if a user generates a model of a coiled coil using the basic building mode, this can be used as a starting point for building an advanced mode model, as the parameters are preserved without restriction.

### Collagen build mode

One major advance of parametric modeling in ISAMBARD, compared to previous software for such modeling of coiled coils, is that the mathematics that describes the parameterization (called a “specification”) is separated from the mathematics that describes the secondary structure. Essentially, in ISAMBARD, the specification contains a mathematical parameterization that creates paths through space, along which secondary structure are built. This means that the coiled‐coil specification, which describes paths that follow a super‐helix, can be equally applied to coiled coils or other helical bundles, such as the collagen triple helix.^46^

As ISAMBARD is used as the modeling engine in CCBuilder 2.0, we have included an option to model the collagen triple helix. Both basic and advanced build modes are available, with parameters for radius, pitch, interface angle, and z‐shift in both modes, with the oligomeric state locked to 3. Both homotypic and heterotypic collagens can be modeled. For the collagen build mode only, the character “O” is also allowed in sequence submissions, which inserts the noncanonical amino acid hydroxyproline into the model at the specified positions.

### Optimizing a model

In contrast to CCBuilder, which requires manual tweaking of parameters to optimize a coiled‐coil model, CCBuilder 2.0 utilizes ISAMBARD to implement the CCScanner protocol, allowing automated optimization of structural parameters. CCBuilder 2.0 optimizes structural parameters based on the BUDE interaction energy between the helices.^49^, ^50^ We have shown previously that this approach can be used to predict the oligomeric state and parameters for a coiled‐coil sequence with high accuracy.^10^ The optimization tab in CCBuilder 2.0 can be used to submit a model for parameter optimization, and, on the server side, uses a Metropolis Monte Carlo optimizer, implemented in the ISAMBARD software package, to improve the values iteratively. Owing to restriction on available server‐side compute, optimizations can only be submitted for models constructed using the basic build mode, and are limited to a maximum of 300 residues. If more‐complex, larger, or high‐throughput optimizations are required, they can be performed on a local computing resource using the ISAMBARD software package directly.

## Conclusions

If carefully balanced, usability of a biomolecular modeling package does not have to be at the expense of modeling power. With CCBuilder, our focus on usability has enabled scores of research scientists, many of whom are nonexpert in atomistic modeling, to produce highly accurate models of coiled coils that can be used for a range of real‐world applications.^52^, ^53^, ^54^, ^55^, ^56^, ^57^, ^58^, ^60^

With CCBuilder 2.0 (http://coiledcoils.chm.bris.ac.uk/ccbuilder2), we have modernized the whole application, preserving the core functionality while adding a range of new features and vastly improving the overall user experience. It is now possible to optimize structural parameters to improve the quality of the model with a single click. Users who wish to dig a little deeper can download the modeling API itself, ISAMBARD,^46^ and perform more complex model‐building routines offline.

Careful consideration has been made to the portability and extendibility of the code base. Heavy users of CCBuilder 2.0, or those with unreliable internet connections, are encouraged to download the application and run it inside a Docker container on their local machine. The application is open source and freely available through GitHub (https://github.com/woolfson-group/ccbmk2). Users are free to modify or extend CCBuilder 2.0, and any useful modifications can be submitted to be merged into the public application, allowing all users to benefit. We plan to update the application frequently in a similar manner, continually adding new features and improving stability, usability, and utility.

A wide range of informatics and modeling tools are becoming available for α‐helical coiled coils.^14^, ^28^, ^29^, ^31^, ^63^, ^64^, ^65^, ^66^ A clear path forward would be to link these tools together to create a unified informatics and modeling service. Such a “CCCentral” facility would allow user to feed data from natural coiled coils directly into design, or structural modeling of putative coiled coils identified using sequence information. We plan to build and present such an environment in the future to benefit a broad swathe of peptide and protein scientists interested in the structural biology, engineering, design, and application of α‐helical coiled coils and related parameterizable protein folds.

## Supporting Information

The Supporting Information associated with this article contains example Python scripts that the web backend uses for generating models.

## Supporting information

Supporting Information
