# Supplementary material for: CCBuilder 2.0: Powerful and accessible coiled‐coil modeling
Source: Protein Sci. 2017 Sep 15;27(1):103–11. doi: 10.1002/pro.3279 (PMC5734305; doi:10.1002/pro.3279)
Supplement: Supplementary file 1 — Supporting Information [file PRO-27-103-s001.pdf]

# CCBuilder 2.0: Powerful and accessible coiled-coil modeling

## Christopher W. Wood and Derek N. Woolfson

## Supplementary Information

### ISAMBARD-Based Model Building Routine

The model building routine used by CCBuilder Mk.2 utilises the ISAMBARD software package to build models, using a simple script that can be run on local machines.

```
"""Module for building models using ISAMBARD."""

import itertools
import sys

import isambard


registerAdjust = {
    'a': 0,
    'b': 102.8,
    'c': 205.6,
    'd': 308.4,
    'e': 51.4,
    'f': 154.2,
    'g': 257
}


def build_coiled_coil(parameters):
    """Builds a model of a coiled coil using the input parameters.

    Parameters
    ----------
    parameter : [ dict[str, int/float/str] ]
        List of parameter dictionaries required for building a coiled coil i.e.
        oligomer state, radius, pitch, phiCA, sequence.

    Returns
    -------
    model_data : dict
        A dictionary containing information about the model that has
        been produced.
    """
    coiled_coil = isambard.specifications.CoiledCoil(len(parameters), auto_build=False)
    coiled_coil.major_radii = [p['Radius'] for p in parameters]
    coiled_coil.major_pitches = [p['Pitch'] for p in parameters]
    raw_phi = [p['Interface Angle'] for p in parameters]
    registers = [p['Register'] for p in parameters]
    coiled_coil.phi_c_alphas = [ia + registerAdjust[r] for ia, r in zip(raw_phi, registers)]
    sequences = [p['Sequence'] for p in parameters]
    coiled_coil.rotational_offsets = [
        d + p['Super-Helical Rotation'] for d, p in zip(
            coiled_coil.rotational_offsets, parameters)]
    coiled_coil.orientations = [-1 if p['Orientation'] else 1 for p in parameters]
    coiled_coil.z_shifts = [p['Z-Shift'] for p in parameters]
    coiled_coil.build()
    coiled_coil.pack_new_sequences(sequences)
    model_data = {
        'pdb': coiled_coil.pdb,
        'mean_rpt_value': calculate_average_rpt(coiled_coil),
        'score': coiled_coil.buff_interaction_energy.total_energy
        }
    return model_data


def optimise_coiled_coil(parameters):
```

```
    """Optimises the parameters for a given structure.

    Parameters
    ----------
    parameter : [ dict[str, int/float/str] ]
        List of parameter dictionaries required for building a coiled coil i.e.
        oligomer state, radius, pitch, phiCA, sequence.

    Returns
    -------
    model_data : dict
        A dictionary containing information about the model that has
        been produced.
    """
    oligomer_state = len(parameters)
    radius_centre = parameters[0]['Radius']
    phi_centre = parameters[0]['Interface Angle'] + registerAdjust[parameters[0]['Register']]
    opt = isambard.optimisation.GA_Opt(
        isambard.specifications.CoiledCoil.from_parameters)
    opt.parameters(
        [parameters[0]['Sequence']] * oligomer_state,
        [radius_centre, 200, phi_centre],
        [2, 100, 20],
        [oligomer_state, len(parameters[0]['Sequence']), 'var0', 'var1', 'var2' ]
        )
    opt.run_opt(20, 5, 2)
    top_model = opt.best_model
    optimised_parameters = {
        'radius' : top_model.major_radii[0],
        'pitch' : top_model.major_pitches[0],
        'phiCA' : top_model.phi_c_alphas[0],
        'sequence' : top_model[0].sequence,
        'register' : parameters[0]['Register']
    }
    model_data = {
        'pdb': top_model.pdb,
        'mean_rpt_value': calculate_average_rpt(top_model),
        'score': top_model.buff_interaction_energy.total_energy
        }
    return { 'parameters' : optimised_parameters, 'modellingResults' : model_data }


def calculate_average_rpt(ampal):
    """Returns the mean residues per turn value for an AMPAL object."""
    rpt_lists = [isambard.analyse_protein.residues_per_turn(ch)[:-1] for ch in ampal]
    rpt_values = list(itertools.chain(*rpt_lists))
    average_rpt = sum(rpt_values)/len(rpt_values)
    return average_rpt
```
